# Impact of the COVID-19 Pandemic on Mental Health Outcomes of Healthy Children, Children With Special Health Care Needs and Their Caregivers–Results of a Cross-Sectional Study

**DOI:** 10.3389/fped.2022.759066

**Published:** 2022-02-10

**Authors:** Anne Geweniger, Michael Barth, Anneke D. Haddad, Henriette Högl, Shrabon Insan, Annette Mund, Thorsten Langer

**Affiliations:** ^1^Center for Pediatrics, Department of Neuropediatrics and Muscle Disorders, Medical Center - University of Freiburg, Freiburg, Germany; ^2^Center for Pediatrics, Department of General Pediatrics, Adolescent Medicine and Neonatology, Medical Center - University of Freiburg, Freiburg, Germany; ^3^Kindernetzwerk e.V., Mainaschaff, Germany

**Keywords:** inequalities, socioeconomic status, mental health, COVID-19, children with special healthcare needs, caregivers, pandemic

## Abstract

**Background:**

Several studies have described widening inequalities as a result of the COVID-19 pandemic, mostly for adult populations. Children and adolescents are particularly impacted by the indirect effects of the pandemic and lockdown measures, such as reduced access to or delays in health care and school closures. National surveys in several countries also show a rising mental health burden in children and adolescents during the COVID-19 pandemic. Children with special health care needs are a particularly vulnerable group in this context as they rely on a wide range of services, which were mostly suspended during the first wave of the pandemic. This study aims: (1) to describe the mental health outcomes of children with and without special healthcare needs and of their caregivers following the first national lockdown in Germany; (2) to investigate variations in mental health outcomes and measures of pandemic burden according to socioeconomic status; (3) to assess the impact of socioeconomic status, disease complexity and psychosocial burden on parent-reported child mental health problems.

**Methods:**

We conducted an online survey among 1,619 caregivers of children aged 1–18 years from August 11th until October 5th 2020. Participants were recruited both from families of children with special healthcare needs and of healthy children. Inequalities were analysed by descriptive statistics, simple and hierarchical logistic regression modelling to explore the association between socioeconomic status and psychological outcome measures, disease complexity and general burden related to COVID-19.

**Results:**

There was a high prevalence of 57.4% of parent-reported mental health problems in children and of a positive screening score for depression in 30.9% of parents. Parent-reported mental health problems were more likely to affect children with low socioeconomic status, with complex chronic disease and those whose parents screened positive for depression.

**Conclusions:**

This study highlights inequalities in parent-reported child mental health outcomes by socioeconomic status and disease complexity in a large sample of German families with and without children with special health care needs. Political measures should put children at the centre and aim to mitigate the unequal impact of the COVID-19 pandemic, particularly on the mental health of vulnerable children.

## Introduction

There is growing concern that COVID-19 widens and exposes pre-existing inequalities (1). Adults of ethnic and minority background, of low socioeconomic status (SES) or those living in deprived neighbourhoods suffer from higher COVID-related morbidity and mortality (2, 3). While inequalities have been described for the adult population, research into pandemic-related inequalities affecting children and adolescents is rare. Measures to control the pandemic, mostly lockdown- and social distancing measures, have lead to a disruption of societal functioning in many countries worldwide. As SARS-CoV2 infection in children leads mostly to mild disease, it is particularly the indirect impact of the pandemic that affects children and adolescents (4–7). These indirect effects are wide-ranging and include reduced access to or delays in health care and immunisation services; educational deficits as a result of school closures; food insecurity; increased risk of child abuse and violence against children; poor mental health and adverse child development outcomes (4, 5, 8–10).

Children and adolescents have been identified as a particularly vulnerable group in this current pandemic, especially with regards to their mental health (11). National surveys in several countries show a rising mental health burden in children and adolescents as a consequence of the COVID-19 pandemic. In Canada, 25% of parents reported worsened child mental health outcomes (12). In the US, an increase in emergency department visits for mental health problems was observed locally and parents reported an increase in child behavioural problems in a national survey (8, 13). In the UK, the proportion of mental health problems increased from 10.8% in 2017 to 16.0% in 2020 in children aged 5–16 years (14). Meanwhile, some studies suggest that there is decreased access to mental health services both due to lower referrals and reduced help-seeking out of fear of contracting SARS-CoV2 (14–16).

In Germany, the first official case of COVID-19 was registered on January 27^th^ 2020. Depending on the respective federal state, schools closed from about the middle of March until May 4^th^ 2020 and a first national lockdown was in place from March 22^nd^ until May 4^th^ 2020. A national representative study, conducted between May 26^th^ and June 10^th^ following the first lockdown found an increase of mental health problems in children and adolescents from 9.9% in 2017 to 17.8% in 2020 and an increase in anxiety from 14.9 to 24.1% (17).

With the daily life of families and children being disrupted by school closures, social distancing and economic consequences of lockdown measures, resulting parental stress and increase in family conflict is likely to have an additional impact on child mental health (7, 11, 12, 18–21). For instance, during the first wave of COVID-19 in 2020 in the UK, children with parents experiencing psychological distress were three times more likely to have mental health problems (14). In Spain, an increase in mental health problems of adolescents was associated with worsened family relationships and stress in the family (21).

The association between low SES and child mental health outcomes has been widely described (12, 17, 22–24). In the current pandemic, children from households in the UK that had fallen behind with payments were more than twice as likely to have mental health problems (14, 15). A study from China reports that both low parental education and income were associated with child mental health problems (25). In Germany, children from a low socioeconomic background were significantly more affected by an increase in mental health problems and anxiety following the first lockdown (17).

Children with special health care needs are a vulnerable population dependent on services relating to health, education and protection which were mostly suspended during the first wave of the pandemic (7). Their caregivers already face a higher mental health burden as well as higher rates of work loss and financial strain due to COVID-19 (26–29). These families and children are particularly affected by disintegration of support networks, as most services required cannot be delivered outside of specialist settings and it is difficult for parents or caregivers to replace the wider support those children usually receive (6, 30). As a consequence, studies suggest that children with special health care needs are facing an increase in mental health problems, delayed access to health care and risk falling even more behind in their education (6, 27, 28, 30–32).

Despite emerging evidence, so far few research has specifically focussed on the impact of inequalities and social determinants on child health in general, and on vulnerable children in particular, during the COVID-19 pandemic (5).

Following calls for “research that unpicks the mechanisms of differential exposure, vulnerability and consequences, identifying the most effective policy entry points” (2), our study aims to investigate the impact of lockdown measures during the first wave of the COVID-19 pandemic in Germany, and of socioeconomic inequalities on the mental health burden of children with and without special health care needs, and of their caregivers. This study aims:

1. to describe the mental health outcomes of children with and without special healthcare needs and of their caregivers following the first national lockdown in Germany.
2. to investigate variations in mental health outcomes and measures of pandemic burden according to socioeconomic status.
3. to assess the impact of socioeconomic status, disease complexity and psychosocial burden on parent-reported child mental health problems.

## Methods

### Study Design

This study represents the first of three rounds of data collection of a sequential cross-sectional study with a survey period of 1.5 years. To this end, an online survey administered through the REDcap© system was conducted from August 11th until October 5th 2020 among caregivers of children aged 1–18 years.

Participants were recruited by convenience and non-probabilistic snowball sampling through the Kindernetzwerk e.V., a patient organisation for families of children with chronic diseases and disabilities, which unites a network of 236 patient organisations with about 200,000 members; through promoting the link on free access websites; and via social und public media. Both families with children with chronic diseases or disabilities, and families with healthy children as comparison group were encouraged to participate in the survey.

The study is registered with the German Registry for Clinical Studies (DRKS00022868) and was approved by the local ethics committee of Freiburg University (Approval number 377/20).

### Measures

#### Socioeconomic Status (SES)

Following the approach of the National Health Interview and Examination Survey for Children and Adolescents (KiGGS) in Germany (33), an index measuring SES was constructed as the sum of three indicators: household net equivalent income, parental education and parental occupation. Household net equivalent income was calculated as the monthly net family income adjusted for household size using a modified scale proposed by the Organisation for Economic Cooperation and Development [OECD, (33)]. Weights were assigned to the household head (=1), any additional adult (=0.5) and children (=0.3). The monthly net family income was divided by the sum of weights per household. For parental education and occupation, the respective higher level of each parent was assigned to each household. The SES index was first divided into quintiles and then grouped into three levels: low (quintile 1), middle (quintiles 2–4) and high (quintile 5) SES, allowing for comparison between the most disadvantaged 20% and the most advantaged 20% of families in the sample.

#### Strengths and Difficulties Questionnaire (SDQ)

The SDQ is an established and validated screening instrument for mental health problems in children and adolescents. The standard parent-reported version of the SDQ applies to children aged 4–16 years (34). The German version of the parent-reported scale has equal validity and reliability compared to the original English version (35–37). Four subscales (hyperactivity/inattention, emotional symptoms, conduct problems, peer problems) are covered by the Total Difficulties Score, which has values from 0–40; higher scores indicate more serious mental health problems. The preschool version of the SDQ differs from the standard parent-reported SDQ in three items, which are adjusted to account for an age-appropriate context (www.sdqinfo.org) (38). The German preschool version has been validated in a sample of 3–5 year old children (39). In line with these previous studies, we used age-appropriate versions of the SDQ for parents of children aged older than 2 years. Following published standards, we used a cut-off of 13 or higher to group slightly raised and high scores as compared to average scores of 0–12 (37, 40). The SDQ relates to child or adolescent behaviour during the previous 6 months and has been used as part of the National Health Interview and Examination Survey for Children and Adolescents (KiGGS) (40).

#### WHO-5 Wellbeing Index (WHO-5)

The WHO-5 is a widely used brief screening questionnaire to assess mental health wellbeing. Its validity and reliability has been documented in various studies and it can be used as a screening tool for depression (41). Referring to the previous 2 weeks, the WHO-5 assesses wellbeing in five questions and a six point Likert-Scale, resulting in a raw score of 0–25. The raw score is multiplied by four to give the final score of 0–100, with 100 representing the best imaginable wellbeing. The cut-off point for depression screening is 50 (41).

#### Children With Special Health Care Needs (CSHCN-Screener)

The CSHCN Screener is a brief standardised survey which aims at identifying children with chronic physical, mental, behavioural or other conditions who require health and related services beyond the average needs of their peers (42). Five parent-reported items assess (1) the need or use of prescription medication; (2) above-routine use of medical, mental health or educational services compared to other children of the same age; (3) activity limitations in day-to-day life compared to similar age children; (4) need or use of specialised therapies; and (5) need or use of treatment or counselling for an emotional, behavioural or developmental condition. A maximum score of 5 can be obtained, with higher scores indicating higher complexity of health care needs and more severe conditions (43). Several ways of stratifying children according to their CSHCN score have been described (43, 44). We categorised children into three groups: healthy (CSHCN score = 0), chronic conditions (CSHCN score ≤2) and complex chronic conditions (CSHCN score ≥3). The CSHCN Screener has been used as part of the National Health Interview and Examination Survey for Children and Adolescents (KiGGS) in Germany (45).

#### COVID-19 and Lockdown-Related Burden

We designed several items assessing the general burden of the impact of the pandemic on families and children, covering financial difficulties, home schooling and child development, social support and a general feeling of being burdened by the pandemic restrictions.

#### Sociodemographic Measures

Sociodemographic measures relating to parents included respondent's age and gender, relationship status and relation to the child, education, occupation, monthly net family income and household size as well as area of residence and country of birth. Measures relating to the child included age and gender.

### Statistical Methods

Descriptive statistics comprised frequencies, means and standard deviations for all variables. Inequalities in the impact of the first wave of the COVID-19 pandemic in Germany were analysed by simple logistic regression to obtain unadjusted Odds Ratios for the association between SES and psychological outcome measures, disease complexity and general burden related to COVID-19 respectively.

Theory-driven hierarchical (blockwise) logistic regression modelling was performed to estimate adjusted Odds Ratios for the association of child mental health problems with SES, disease complexity, parental mental health and pandemic burden. Block 1 represents associations between child mental health problems, gender, disease complexity and SES, which have been widely described. Block 2 represents potential associations between parental wellbeing during the pandemic and child mental health problems. Block 3 represents additional COVID-19 related burden for both parents and children. A general model (model 1) was compared with three models stratifying for disease complexity to control for confounding (models 2–4). The analysis was restricted to children older than 2 years of age, in accordance with the SDQ age limit as outlined above:

**Model 1:**
  - block 1: child's age, gender, socioeconomic status, disease complexity
  - block 2: WHO-5
  - block 3: increase in family conflict, financial difficulties, inadequate educational support, negative effect of school closures on child's development, inadequate social support, area of residence
**Model 2:** Model 1 restricted to healthy children.
**Model 3:** Model 1 restricted to children with chronic conditions.
**Model 4:** Model 1 restricted to children with complex chronic conditions.

The overall model fit was assessed by R^2^ statistics. Model sensitivity and R^2^ statistics are reported for each model. Adjusted Odds Ratios are reported with the corresponding 95% confidence intervals (95% CI). Prior to logistic regression modelling, we replaced missing data of outcome and exposure variables using multiple linear imputation to include all cases (*N* = 1,619). Therefore, we proved the missing at random assumption by including all variables of the logistic regression model and *COVID-19 and lockdown-related burden* variables. Missing values ranged from 15–24% for all variables.

Analysis was conducted using IBM SPSS Version 27.0.

## Results

### Sociodemographic Characteristics

Of 2004 persons who accessed the survey, 1,619 had children aged 18 years or younger, thus meeting the inclusion criteria.

The mean age of respondents was 41 years (SD 6.94) and 90.1% (*n* = 1,458) were female. The mean age of children was 8 years (SD 4.19) and 55.5% (*n* = 899) were male. The majority lived with their biological parents in the same household (Table 1). In 84.3% (*n* = 1,071) of cases both parents were born in Germany. Most parents had a high educational level and worked in full-time or free-lance jobs (*n* = 1,173 [72.5%] and *n* = 1,214 [75.0%] respectively). Among children, 29.5% (*n* = 478) fulfilled the criteria for complex chronic disease according to the CSHCN Screener, 11.9% (*n* = 193) for chronic disease and the majority (58.6%, *n* = 948) was healthy.

**Table 1:** Sociodemographic characteristics (*N* = 1619).

	**Mean (SD)**	**Range**
Age in years		
Responding parent	41.2 (6.94)	
All children	8.14 (4.19)	1–19
Children with SHCN	8.99 (4.27)	0–18
Number of children per household	1.95 (0.83)	1–7
Household size	3.98 (0.99)	1–9
	***n***	**%**
Gender of respondent		
Male	161	9.9
Female	1,458	90.1
Gender of child		
Male	899	55.5
Female	710	43.9
Diverse	10	0.6
Relation to child (*N* = 1,307)		
Biological mother	1,136	86.9
Biological father	125	9.6
Other	46	3.5
Relationship status		
With partner, in same household	1,441	89.0
With partner, not in same household	46	2.8
No partner	132	8.2
Country of birth of parents (*N* = 1,271)		
Both in Germany	1,071	84.3
One parent in Germany, one elsewhere	154	12.1
Both elsewhere	46	3.6
Place of residence		
City (>100,000 inhabitants)	558	34.5
Surroundings of a city with >100,000 inhabitants	226	14.0
Town (20,000- 100 000 inhabitants)	238	14.7
Small town (5,000 – 20,000 inhabitants)	235	14.5
Rural municipality (<5,000 inhabitants)	362	22.4
Disease complexity of child		
Healthy (CSHCN = 0)	948	58.6
Chronic disease (CSHCN ≤ 2 criteria)	193	11.9
Complex chronic disease (CSHCN ≥ 3 criteria)	478	29.5
Educational level		
No qualification	1	0.1
Certificate of Secondary Education (9 years of schooling)	36	2.2
General Certificate of Secondary Education (10 years of schooling)	409	25.3
A-levels (qualification at age 18)	1,173	72.5
Employment status		
Inactive or unemployed	30	1.9
Maternity leave or parental leave	23	1.4
Short term or temporary employment	98	6.1
Part-time	254	15.7
Full-time or free lance	1,214	75.0
Household net equivalent income (*monthly, in Euro, quintiles*)		
Up to 850	368	22.7
850–1000	312	19.3
1000–1300	314	19.4
1300–1400	364	22.5
More than 1,400 *(maximum: 2,750 €)*	261	16.1

*SD, Standard deviation; percentages reported as row percentages*.

Among all 671 children with special health care needs, 69.4% (*n* = 466) had a physical impairment, 73.0% (*n* = 490) had a behavioural or sensory impairment, and 53.7% (*n* = 360) had impaired speech or understanding. Difficulties in communication were reported for 27.8% (*n* = 188) children, 1.6% (*n* = 11) depended on home ventilation and 5.6% (*n* = 38) had a feeding tube.

### Mental Health and Perceived Burden of the Pandemic

For parents, the mean WHO-5 score was 62.8 (SD 20.8), with 30.9% (*n* = 501) having a score below the screening cut-off for depression. For children, the parent-reported mean SDQ-score was 13.5 (SD 5.2), with 57.4% (*n* = 930) of children having a score at or above the cut-off level of 13, indicating slightly raised or high scores. There was very strong evidence for an association of the SDQ score and disease complexity (χ^2^(df=2) = 104.06; *p* < 0.001). Among healthy children, 52.8% (*n* = 501) had average SDQ scores, whereas a majority of children with chronic and complex chronic disease had slightly raised or high SDQ-scores (*n* = 126 [65.3%] and *n* = 357 [74.7%] respectively). Most parents felt burdened by the pandemic restrictions (*n* = 1,445 [89%]) and saw their children burdened as well (*n* = 1,426 [88.1%]). About half the parents felt burdened by home schooling, perceived the educational support as inadequate and saw a negative effect of school closures on their child's development (Table 2). More than one third of parents reported an increase in family conflict and felt that social support by friends or relatives was inadequate (*n* = 623 [38.5%] and *n* = 563 [34.8%] respectively).

**Table 2:** Negative effects of the first lockdown on families; *N* = 1,619.

	***n***	**%**
Increase in family conflict	623	38.5
Inadequate social support	563	34.8
Financial difficulties due to the pandemic	268	16.6
Feeling burdened by home schooling	937	57.9
Inadequate educational support	857	52.9
Negative effect of school closures on child's development	807	49.8
Feeling burdened by pandemic restrictions		
Respondent	1,445	89.3
Child or children	1,426	88.1
Child with chronic condition (*N* = 515)	418	81.2

### Socioeconomic Inequalities

**Figure 1:**
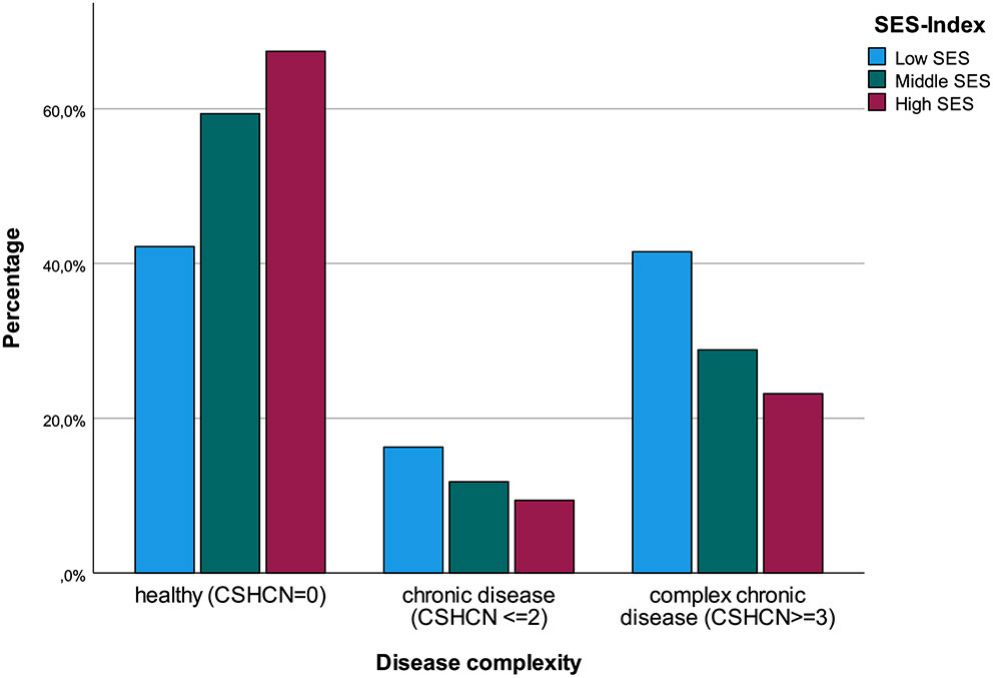
Stratified analysis of disease complexity by socioeconomic status (*N* = 1619). SES, socioeconomic status; CSHCN, Children with Special Health Care Needs Screener.

Stratifying the sample according to the SES index showed that 18.6% (*n* = 301) of all families had a low SES, 51.8% (*n* = 839) were in the middle group, whereas 29.6% (*n* = 479) had a high SES. There were marked differences between families of low and high SES. There was strong evidence for an unequal distribution of disease complexity among children in different socioeconomic groups, with more children with complex chronic conditions living in families of low socioeconomic status (χ^2^(df=4) = 49.04; *p* < 0.001; Figure 1 and Supplementary Table S1). Looking at mental health outcomes, parents of low SES had 86% higher odds of a score suggestive of depression in the WHO-5 screening than parents with a high SES (crude OR 1.86 [1.37; 2.54]; *p* < 0.001; Table 3). Similarly, children living in families with a low SES had twice the odds of parent-reported mental health problems compared to children in the highest SES group (crude OR 2.32 [1.71; 3.14]; *p* < 0.001). Families with a low socioeconomic status had seven times the odds of financial difficulties compared to families in the highest group and had 90% higher odds of reporting inadequate social support (crude OR 7.03 [4.59; 10.77]; *p* < 0.001 and crude OR 1.90 [1.41; 2.57]; *p* < 0.001). There was weak evidence for a difference in the burden of homeschooling and increase of family conflict between the respective SES groups (Table 3). No differences were observed for educational support and the negative effect of school closures on the child's development.

**Table 3:** Stratified analysis of psychosocial outcomes and perceived burden by socioeconomic status (*N* = 1619).

	**Low SES**	**Middle SES**	***p*-value** **(reference group:** **High SES)**
	**OR (95% CI)**	**OR (95% CI)**	
WHO-5 ≤ 50	1.86 (1.37; 2.54)	1.33 (1.03; 1.71)	<0.001
SDQ total score ≥13	2.32 (1.71; 3.14)	1.39 (1.11; 1.74)	<0.001
Increase in family conflict	1.41 (1.05; 1.89)	1.17 (0.93; 1.48)	0.075
Inadequate social support	1.90 (1.41; 2.57)	1.23 (0.97; 1.57)	<0.001
Financial difficulties	7.03 (4.59; 10.77)	2.52 (1.69; 3.76)	<0.001
Inadequate educational support	1.14 (0.85; 1.52)	1.06 (0.85; 1.33)	0.673
Feeling burdened by home schooling	1.26 (0.94; 1.69)	1.30 (1.04; 1.64)	0.063
Negative effect of school closures on child's development	1.05 (0.78; 1.40)	1.19 (0.95; 1.49)	0.278

*Unadjusted Odds Ratios (OR) are estimated by logistic regression modelling. SES, socioeconomic status; CSHCN, Children with Special Health Care Needs Screener; WHO-5, WHO-5 Wellbeing Index, SDQ, Strengths and Difficulties Questionnaire*.

### Impact of Socioeconomic Status and Psychosocial Burden on Child Mental Health

The results from hierarchical logistic regression modelling are displayed in Table 4 for the baseline model and in the Supplementary Table S2 for models 2–4.

**Table 4:** Impact of socioeconomic status and psychosocial burden on parent-reported child mental health problems.

**Variables**	**Model 1**		
	**All children**		
	**(*****N*** **=** **1,479)**		
**Block 1**	**OR**	**95% CI**	***p***
*Disease complexity*			
Healthy (referent)			
Chronic condition	**2.34**	1.62; 3.37	<0.001
Complex chronic condition	**3.07**	2.33; 4.04	<0.001
*SES-Index*			
Low	**1.65**	1.14; 2.38	0.007
Middle	1.27	0.97; 1.66	0.084
High (referent)			
Age of child	**0.96**	0.93; 0.99	0.004
*Gender of child*			
Male (referent)			
Female	0.82	0.65; 1.04	0.095
Diverse	2.55	0.46; 14.06	0.283
**Model fit after block 1**			
Nagelkerke's R^2^	0.13		
**Block 2**	**OR**	**95% CI**	***p***
WHO-5 Score ≤50	**1.91**	1.47; 2.49	<0.001
**Model fit after block 2**			
Nagelkerke's R^2^	0.19		
Δ R^2^	0.05		
**Block 3**	**OR**	**95% CI**	***p***
Increase in family conflict	**2.05**	1.59; 2.62	<0.001
Financial difficulties	**1.60**	1.15; 2.23	0.006
Inadequate educational support	**1.72**	1.33; 2.22	<0.001
Negative effect of school closures on child's development	**1.39**	1.09; 1.78	0.008
Inadequate social support	**1.39**	1.07; 1.80	0.013
*Area of residence*			
City (referent)			
Surroundings of a city	1.21	0.84; 1.74	0.315
Town	**1.67**	1.16; 2.42	0.006
Small town	**1.46**	1.02; 2.10	0.039
Rural municipality	1.32	0.96; 1.82	0.087
**Model fit after block 3**			
Nagelkerke's R^2^	0.28		
Δ R^2^	0.09		
% correct prediction of SDQ Score ≥13	75.0		

*Hierarchical logistic regression modelling of the outcome SDQ total score ≥13 in children older than 2 years of age. Adjusted Odds Ratios with p < 0.05 in bold*.

*SDQ, Strengths and Difficulties Questionnaire; CSHCN, Children with Special Health Care Needs Screener; SES-Index, Index of socioeconomic status*.

The baseline **model 1** included all children older than 2 years (34). The variance explained by model 1 after the first block including disease complexity, SES index, age and gender of the child was R^2^ = 0.13. The biggest increase in explained variance was after block 3, which included psychosocial explanatory variables (R^2^ = 0.28 and Δ R^2^ =0.09). After completion of block 3, the baseline model 1 suggests very strong evidence for an association of the child's disease complexity, low socioeconomic status, parental mental health and psychosocial burden of the COVID-19 pandemic with parent-reported child mental health problems. After controlling for confounding effects of age, gender and socioeconomic status, children with complex chronic diseases had 3 times the odds of parent-reported mental health problems (i.e., SDQ total score ≥ 13) compared with healthy children in the baseline group (adjusted OR 3.07 [2.33; 4.04]; *p* < 0.001). Children of low socioeconomic status or from families with financial difficulties had 60% higher odds of parent-reported mental health problems compared to children from a high socioeconomic background (adjusted OR 1.65 [1.14; 2.38] and 1.60 [1.15; 2.23] respectively). Children whose parents screened positive for depression in the WHO-5 or reported an increase in family conflict due to the pandemic had twice the odds of parent reported mental health problems compared to the baseline group (adjusted OR 1.91 [1.47; 2.49] and 2.05 [1.59;2.62]; *p* < 0.001 respectively). Area of residence in a town or small town was associated with higher odds of parent-reported mental health problems (adjusted OR 1.67 [1.16; 2.42] and 1.46 [1.02; 2.10]). Conversely, older children had lower odds of a total SDQ-score ≥ 13 (adjusted OR 0.96 [0.93; 0.99]; *p* = 0.004).

**Model 2** for healthy children explained less variance after block 1 (R^2^ = 0.05) than model 1. Similarly, the biggest difference in explained variance was between block 2 and 3 (R^2^ = 0.22 and Δ R^2^ =0.12), however the overall variance explained was lower than for model 1. Model 2 still shows very strong evidence for an association of parental mental health, increase in family conflict, consequences of school closures and strong evidence for an association of low SES with parent-reported child mental health problems. Financial difficulties too were still associated with parent-reported child mental health problems, but not as strongly as in model 1 (adjusted OR 1.58 [1.02; 2.45], *p* = 0.40).

The variance explained by **model 3** including children with less complex chronic disease after block 1 was R^2^ = 0.10. Overall, the explained variance increased only to R^2^ = 0.19 after block 3, with Δ R^2^ =0.04 after block 2 and Δ R^2^ =0.06 after block 3. The model suggests strong evidence for an inverse association of age and parent-reported mental health problems 1 (adjusted OR 0.88 [0.80; 0.98], *p* = 0.014). Apart from weak evidence of an association with parental mental wellbeing (adjusted OR 2.10 [0.98; 4.48]; *p* = 0.056), none of the other exposure variables included in the model show a significant association with the outcome.

Following block 1, the variance explained by **model 4** for children with complex chronic diseases was the lowest of all four models at R^2^ = 0.03. However, it also had the largest increases in explained variance after blocks 2 and 3 of all four models (Δ R^2^ = 0.08 after block 2 and Δ R^2^ = 0.12 after block 3). Overall, the variance explained after block 3 was R^2^ = 0.23. Looking at mental health outcomes in children with complex chronic diseases, model 4 shows a higher influence of psychosocial variables on child mental health than the other models. Here, parents who screen positive for depression had 2.3 times the odds of reporting child mental health problems (adjusted OR 2.35 [1.36; 4.05], *p* = 0.002). Similarly, children whose parents report an increase in family conflict have 2.4 times the odds of parent-reported mental health problems compared to the baseline group (Table 4). Some weak evidence suggestive of an association of low SES with mental health problems remains (adjusted OR 1.99 [0.93; 4.01], *p* = 0.056). However, in this model stratified according to disease complexity, financial difficulties and age show no association with parent-reported child mental health problems anymore.

## Discussion

This is to our knowledge the first study reporting inequalities in mental health outcomes in a large sample of children with and without special health care needs and their parents in Germany. We found a high prevalence of 57.4% of parent-reported mental health problems in children and a positive screening score for depression in 30.9% of parents. Parent-reported mental health problems were more likely to affect children from low SES, with complex chronic disease and those whose parents screened positive for depression.

### Child and Parental Mental Health

Our results suggest a high mental health burden during the pandemic in both children and their families, particularly mothers. The prevalence of parent-reported child mental health problems in our sample was 57.4%. This is a markedly higher prevalence than the prevalence of 30.4% after the first lockdown in the representative German study by Ravens-Sieberer et al. using the same SDQ cut off values as our study (23). However, our study is not based on a representative sample and the high proportion of children with chronic disease might lead to a higher prevalence of child mental health problems in our sample than in the general population.

The mean WHO-5 Score as indicator of wellbeing in our study was 62.8, which is slightly lower than the referent mean score of 65.7 reported for German adults in the European Quality of Life survey [(41), supplement]. 30.9% of parents had a WHO-5 score below the cut off for depression. The European Health Interview Survey 2014/2015 reports for Germany a prevalence of depression of 9.2% in the general population and 10.8% for women (46). Though not resulting from a representative sample, our data suggests both lower parental wellbeing and a higher prevalence of depression compared to the general population. The higher mental health impact of the pandemic on parents has also been described in a representative study from Canada, where 44.3% of parents reported deteriorated mental health compared to 35.6% of adults without children <18 years at home (12).

Our data suggests that children whose parents screened positive for depression in the WHO-5 had twice the odds of parent-reported mental health problems compared to the baseline group. The association of parental and child mental health has been widely acknowledged (9, 47). During the current pandemic, 10% of US families reported both worsening of their own and their child's mental health (8). An Italian survey described the reciprocal relationship of parental psychological distress and child emotional and behavioural problems, especially for single parents or parents of younger children (48). The authors highlight the risk of a vicious circle where parental psychological distress is maintained by children's emotional and behavioural difficulties and aggravates those difficulties in turn.

Similarly, an increase in family conflict was associated with higher odds of parent-reported child mental health problems. This echoes findings from a representative UK study conducted during the first wave of the COVID-19 pandemic. They found that 28.3% of children with mental health problems lived in a family which reported problems with family functioning, compared to 11.7% of children without mental health problems (14). The same study also reported that children with parent-reported mental health problems were less likely to have regular support from their school or college (14). Our findings support this association, as both insufficient social and educational support were associated with parent-reported child mental health problems.

### Socioeconomic Inequalities and Mental Health

We found that children of low socioeconomic status and children from families with financial difficulties had 60% higher odds of parent-reported mental health problems, compared with children from a high socioeconomic background or from families without financial difficulties. Children from lower socioeconomic background have repeatedly been found to be more likely to have mental health problems (22, 24). This inequality persists in the current pandemic and our results seem to be in line with a rise in child and adolescent mental health problems in Germany, which was found to disproportionately affect children whose parents had a lower educational or migration background (17, 23). Similarly, a study conducted in a sample of hourly service workers with young children in the US reports an association of income or job loss, parental and child psychological wellbeing. The number of pandemic-related hardships was related to all psychological well-being measures (20).

The association between low SES and parent-reported child mental health problems remained strong after stratifying the sample according to disease complexity to account for potential confounding. Following stratification, children without complex chronic disease from a low SES had 73% higher odds of parent-reported mental health problems compared with children from a high socioeconomic background. In addition, there was suggestive evidence that children with complex chronic disease from a low SES have even twice the odds of parent-reported mental health problems compared to children from a higher SES. Low SES has been acknowledged to likely be both a cause and a consequence of childhood disability, though its position on the causal pathway remains uncertain (49). A systematic review and meta-analysis by Spencer and colleagues reports higher odds of all-cause disabling chronic conditions for children from low SES (49). Children with complex chronic conditions from a low socioeconomic background might thus be a particularly vulnerable group, concerning both their chronic condition and their mental health. Following priorities set out by the UN ‘Research Roadmap for the COVID-19 Recovery', future research should investigate into potential new forms of vulnerability as a result of the pandemic, taking the complex interaction of multiple coexisting factors into account with the aim to identify measures to address the systemic causes of socioeconomic inequities in health (50).

### Disease Complexity and Mental Health

Children with complex chronic disease had three times the odds of parent-reported mental health problems compared to healthy children in the baseline model. In the stratified analysis by disease complexity, the odds of mental health problems by both a positive screening for depression in the WHO-5 and family conflict increased with the degree of disease complexity. Children with complex chronic disease whose parents screened positive for depression or reported an increase in family conflict had more than two times the odds of parent-reported mental health problems compared to the baseline group.

In our sample, children with a variety of conditions were included, among them behavioural problems, physical and cognitive impairment as well as children with home-ventilation. It is likely that for some of the children with high disease complexity behavioural or emotional problems are part of their condition.

However, the interplay of child and parental mental health warrants further consideration. Parents of children with chronic conditions face a higher risk of psychological distress and depression (27, 48). Several studies have explored the additional mental health burden of carers of children with chronic conditions and disabilities during the COVID-19 pandemic. Willner et al. report moderate to severe levels of depression in 45% of carers of children with intellectual disability (51). Bentenuto et al. investigated factors determining the psychological impact of lockdown measures on parents of children with neurodevelopmental disorders. They report both an increase in parental stress as well as an increase in child behavioural problems (27). Grumi et al. describe the effect of discontinued rehabilitation services for children with neurodevelopmental disorders on the mental health response of caregivers. They found elevated parental scores for stress, anxiety and depression as a consequence of managing the specific special needs of their children without external support (28). The high odds of mental health problems in children with complex chronic disease in our study sample might thus, as a parent-reported outcome measure, equally reflect on parental mental health considering the reciprocity of the relationship.

## Limitations

Despite the large number of participants, the results and in particular the generalisability are limited by the non-representative nature of our sample. In addition, due to the snowballing process of recruitment we do not know exactly through which channels the comparison group of healthy children was reached. Moreover, a majority of respondents had a high education level, whereas lower education and occupational levels are underrepresented leading to responding bias. Furthermore, the cross-sectional design does not allow inference of causality with regards to the main outcome measures. As mentioned above, the presence of parental mental health problems may be reflected in child mental health problems, but child mental health problems may also affect parental mental health.

Regarding statistical analyses, the percentage of missing values ranged from 15–24% for all variables. Instead of restricting the analysis to complete responses with a risk of selection bias and loss of precision, we decided to perform multiple linear imputation for outcome and exposure variables prior to conducting hierarchical logistic regression modelling. However, multiple imputation cannot neutralise measurement errors completely and thus is a compromise in dealing with missing data. Though following an established methodology (33), the construction of the SES-index as a composite measure of socioeconomic status might be a source of bias. The household net income, from which the net equivalent income was calculated as one dimension of the SES-index, was only available as a categorical and not as a continuous variable. The weighting by household size was thus performed on the respective income categories and the actual net equivalent income was then inferred from the income ranges assigned to the original categories. Therefore the potential for under- or overestimating the actual household net equivalent income is high, which also impacts the validity of the SES index.

## Conclusion

This study highlights inequalities in parent-reported child mental health outcomes by socioeconomic status and disease complexity in a large sample of German families with and without children with special health care needs. It shows a high burden of both child and parental mental health problems in the whole study population and identifies children from low SES with complex chronic disease as a particularly vulnerable group concerning the effect of pandemic measures on their mental health outcomes. COVID-19 has already been described as a syndemic, a term which acknowledges the complex interaction of pre-existing conditions, social and biological factors and calls for an integrated approach in response to the pandemic (52, 53). Long-term policies, which put children at the centre, aim at reducing health inequalities, and mitigate the unequal impact of the pandemic, should be an integral part of the COVID-19 response.

## Data Availability Statement

The raw data supporting the conclusions of this article will be made available by the authors, without undue reservation.

## Ethics Statement

This study was reviewed and approved by the Ethics Committee of Freiburg University (Approval number 377/20). The participants provided their written informed consent to participate in this study.

## Author Contributions

MB, TL, and AH conceptualised the study. AG, MB, and TL developed the methodology. TL, AM, and HH recruited participants. AG and MB conducted data analysis. AG, MB, and SI were responsible for data curation. AG wrote the original draft. AG, MB, and TL edited and reviewed the manuscript. MB and TL provided project supervision. TL was in charge of project administration and funding acquisition. All authors contributed to the article and approved the submitted version.

## Funding

This research was funded by the Volkswagen Foundation, grant number Az 990622. The article processing charge was funded by the Baden-Wuerttemberg Ministry of Science, Research and Art and the University of Freiburg in the funding programme Open Access Publishing.

## Conflict of Interest

The authors declare that the research was conducted in the absence of any commercial or financial relationships that could be construed as a potential conflict of interest.

## Publisher's Note

All claims expressed in this article are solely those of the authors and do not necessarily represent those of their affiliated organizations, or those of the publisher, the editors and the reviewers. Any product that may be evaluated in this article, or claim that may be made by its manufacturer, is not guaranteed or endorsed by the publisher.
