# Supplementary material for: Impact of the COVID-19 Pandemic on Mental Health Outcomes of Healthy Children, Children With Special Health Care Needs and Their Caregivers–Results of a Cross-Sectional Study
Source: Front Pediatr. 2022 Feb 10;10:759066. doi: 10.3389/fped.2022.759066 (PMC8866820; doi:10.3389/fped.2022.759066)
Supplement: Supplementary file 1 [file Data_Sheet_1.PDF]

Supplementary Material

Table S1: Stratified analysis of disease complexity by socioeconomic status (N=1619) Estimation by Pearson's Chi Square Statistics. **SES**: socioeconomic status; **df**: degrees of freedom; percentages given as column percentages; **CSHCN**: Children with Special Health Care Needs Screener**.**

	**Low SES**	**Middle SES**	**High SES**			
	n(%)	n(%)	n(%)	Chi^2	df	p-value
Disease complexity of child				49.04	4	<0.001
healthy (CSHCN= 0)	127 (42.2)	498 (59.4)	323 (67.4)			
Chronic disease (CSHCN ≤ 2 criteria)	49 (16.3)	99 (11.8)	45 (9.4)			
Complex chronic disease (CSHCN ≥ 3 criteria)	125 (41.5)	242 (28.8)	111 (23.2)			

Table S2: Impact of socioeconomic status and psychosocial burden on child mental health problems
Hierarchical logistic regression modelling of the outcome SDQ total score ≥13 in children older than 2 years of age. Adjusted Odds Ratios and the corresponding 95% confidence interval (95% CI) are reported for associations of the outcome and the respective exposure variable. Adjusted Odds Ratios with p-value <0.05 in bold. **SDQ**: Strengths and Difficulties Questionnaire; **CSHCN**: Children with Special Health Care Needs Screener; **SES-Index**: Index of socioeconomic status.

**Variables**	**Model 1**			**Model 2**			**Model 3**			**Model 4**		
	all children (N=1479)			CSHCN=0 (N=857)			CSHCN≤2 (N=181)			CSHCN≥3 (N=441)		
**Block 1**	**OR**	**95% CI**	**p**	**OR**	**95% CI**	**p**	**OR**	**95% CI**	**p**	**OR**	**95% CI**	**p**
Disease complexity												
Healthy (referent)												
Chronic condition	**2.34**	1.62; 3.37	<0.001									
Complex chronic condition	**3.07**	2.33; 4.04	<0.001									
SES-Index												
Low	**1.65**	1.14; 2.38	0.007	**1.73**	1.06; 2.83	0.029	0.91	0.31; 2.64	0.857	1.99	0.93; 4.01	0.056
Middle	1.27	0.97; 1.66	0.084	**1.42**	1.02; 2.00	0.041	0.55	0.23; 1.32	0.18	1.29	0.73; 2.28	0.37
High (referent)												
Age of child	**0.96**	0.93; 0.99	0.004	**0.95**	0.91; 0.99	0.011	**0.88**	0.80; 0.98	0.014	1.00	0.95; 1.07	0.886
Gender of child												
Male (referent)												
Female	0.82	0.65; 1.04	0.095	0.79	0.58; 1.06	0.117	0.56	0.28; 1.11	0.094	1.06	0.65; 1.73	0.815
Diverse	2.55	0.46; 14.06	0.283	5.48	0.55; 54.93	0.148				0.23	0.02; 2.06	0.27
**Model fit after block 1**												
Nagekerke's R^2	0.13			0.05			0.10			0.03		
**Block 2**	**OR**	**95% CI**	**p**	**OR**	**95% CI**	**p**	**OR**	**95%**	**p**	**OR**	**95%**	**p**
WHO-5 Score ≤50	**1.91**	1.47; 2.49	<0.001	**1.69**	1.20; 2.38	0.003	2.10	0.98; 4.48	0.056	**2.35**	1.36; 4.05	0.002
**Model fit after block 2**												
Nagekerke's R^2	0.19			0.10			0.14			0.11		
Δ R^2	0.05			0.05			0.04			0.08		
**Block 3**	**OR**	**95% CI**	**p**	**OR**	**95% CI**	**p**	**OR**	**95% CI**	**p**	**OR**	**95% CI**	**p**
Increase in family conflict	**2.05**	1.59; 2.62	<0.001	**2.19**	1.59; 3.01	<0.001	1.36	0.66; 2.83	0.40	**2.41**	1.43; 4.05	0.001

Financial difficulties	**1.60**	1.15; 2.23	0.006	**1.58**	1.02; 2.45	0.040	1.33	0.52; 3.41	0.548	1.61	0.82; 3.13	0.166
Inadequate educational support	**1.72**	1.33; 2.22	<0.001	**1.64**	1.17; 2.29	0.004	1.85	0.92; 3.73	0.084	**1.86**	1.13; 3.07	0.015
Negative effect of school closures on child's development	**1.39**	1.09; 1.78	0.008	**1.49**	1.09; 2.04	0.013	0.91	0.45; 1.85	0.792	1.61	0.98; 2.65	0.059
Inadequate social support	**1.39**	1.07; 1.80	0.013	**1.41**	1.01; 1.99	0.047	1.28	0.58; 2.83	0.549	1.64	0.99; 2.71	0.057
Area of residence												
City (referent)												
Surroundings of a city	1.21	0.84; 1.74	0.315	1.24	0.79; 1.95	0.36	1.94	0.59; 6.31	0.280	0.97	0.45; 2.09	0.944
Town	**1.67**	1.16; 2.42	0.006	**1.90**	1.17; 3.09	0.009	2.10	0.72; 6.19	0.177	1.30	0.64; 2.63	0.464
Small town	**1.46**	1.02; 2.10	0.039	1.36	0.86; 2.17	0.194	1.40	0.50; 3.97	0.524	1.78	0.82; 3.89	0.15
Rural municipality	1.32	0.96; 1.82	0.087	1.42	0.94; 2.15	0.093	1.01	0.39; 2.59	0.988	1.34	0.70; 2.55	0.38
**Model fit after block 3**												
Nagelkerke's R^2	0.28			0.22			0.19			0.23		
ΔR^2	0.09			0.12			0.06			0.12		
% correct prediction of SDQ Score ≥13	75.0			57.9			83.2			91.6		
